# A Direct Comparison of Patients With Hereditary and Sporadic Pancreatic Neuroendocrine Tumors: Evaluation of Clinical Course, Prognostic Factors and Genotype–Phenotype Correlations

**DOI:** 10.3389/fendo.2021.681013

**Published:** 2021-05-28

**Authors:** Przemysław Soczomski, Beata Jurecka-Lubieniecka, Aleksandra Krzywon, Alexander Jorge Cortez, Stanisław Zgliczynski, Natalia Rogozik, Małgorzata Oczko-Wojciechowska, Agnieszka Pawlaczek, Tomasz Bednarczuk, Barbara Jarzab

**Affiliations:** ^1^ Department of Nuclear Medicine and Endocrine Oncology, Maria Sklodowska-Curie National Research Institute of Oncology, Gliwice Branch, Gliwice, Poland; ^2^ Department of Biostatistics and Bioinformatics, Maria Sklodowska-Curie National Research Institute of Oncology, Gliwice Branch, Gliwice, Poland; ^3^ Department of Internal Diseases and Endocrinology, Medical University of Warsaw, Warsaw, Poland; ^4^ Laboratory of Molecular Diagnostics and Functional Genomics, Department of Nuclear Medicine and Endocrine Oncology, Maria Sklodowska-Curie National Research Institute of Oncology, Gliwice Branch, Gliwice, Poland

**Keywords:** multiple neuroendocrine neoplasia type 1, *V*on Hippel–Lindau Syndrome, pancreatic neuroendocrine tumor, hereditary pancreatic neuroendocrine tumor (PNET)******, comparison, genotype-phenotype correlation

## Abstract

**Introduction:**

Pancreatic neuroendocrine tumors (PNETs) in hereditary syndromes pose a significant challenge to clinicians. The rarity of these syndromes and PNETs itself make it difficult to directly compare them with sporadic PNETs. Despite research suggesting differences between these two entities, the same approach is used in hereditary and sporadic PNETs.

**Methods:**

We included 63 patients with hereditary PNET (GpNET) and 145 with sporadic PNET (SpNET) in a retrospective observational study. Clinical and genetic data were collected in two Polish endocrine departments from January 2004 to February 2020. Only patients with confirmed germline mutations were included in the GpNET cohort. We attempted to establish prognostic factors of metastases and overall survival in both groups and genotype–phenotype correlations in the GpNET group.

**Results:**

Patients with GpNET were younger and diagnosed earlier, whereas their tumors were smaller and more frequently multifocal compared with patients with SpNET. Metastases occurred more frequently in the SpNET group, and their appearance was associated with tumor size in both groups. GpNET patients had longer overall survival (OS). OS was affected by age, age at diagnosis, sex, grade, stage, tumor diameter, occurrence and localization of metastases, type of treatment, and comorbidities. In the MEN1 group, carriers of frameshift with STOP codon, splice site, and missense mutations tended to have less advanced disease, while patients with mutations in exon 2 tended to have metastases more frequently.

**Conclusions:**

Direct comparisons of GpNET and SpNET demonstrate significant differences in the clinical courses of both entities, which should force different approaches. A larger group of patients with GpNET should be assessed to confirm genotype–phenotype correlations.

## Introduction

Pancreatic neuroendocrine tumors (PNETs) are a rare type of neoplasms that constitute approximately 1–2% of all pancreatic cancers. The incidence of PNETs has substantially increased in recent years and is now estimated at 0.01–0.8 cases per 100,000 per year (1–3). It is probably caused by improvements in diagnostics and increased awareness of NET (3). Yet, some authors claim that the incidence of PNETs could still be underestimated (4). PNETs account for approximately 30% of all gastroenteropancreatic neuroendocrine tumors (GEPNETs), and the majority of them are non-functional (NFpNET) (5). Life expectancy in PNETs is lower than in the general population and varies with many factors (6). Additionally, it has the worst median overall survival among all the NET locations (7).

Less than 10% of PNETs arise in the context of familial syndromes. Hereditary syndromes associated with PNETs are Multiple Endocrine Neoplasia type 1 (MEN1), Von Hippel–Lindau disease (VHL), Neurofibromatosis type 1 (NF1), and Tuberous Sclerosis Complex (TSC). The prevalence of PNETs in these syndromes is 30–80% in MEN1 (8), 12% in VHL (9), less than 1% in NF1 (10), and approximately 2% in TSC (11). The disease course in syndromes associated with germline mutations varies from the sporadic tumors. Because of the sparsity of PNETs in NF1 and TSC, we have most of the data coming from the analysis of the MEN1 and VHL populations. PNETs observed in these syndromes are usually diagnosed earlier and have multifocal presentations with an indolent disease course (12–14). Moreover, MEN1-related PNETs are known to be the main reason for syndrome-related deaths (15, 16). Regarding hormone production, gastrinoma is the most frequent functional tumor in MEN1, whereas insulinoma dominates in TSC (17, 18). It appears PNETs in VHL syndrome are not functional (9, 13).

Studies that directly compare cohorts of patients with familial and sporadic PNETs are scarce (13, 14, 19, 20). All the groups confirmed the differences in the clinical features of MEN1/VHL-related PNETs. Moreover, Chiloiro et al. showed no significant differences in prognosis between MEN1-related PNET and sporadic ones, whereas Demestier demonstrated better outcomes in the postoperative course of patients with VHL-related PNET (14, 19). There are no data on such comparisons regarding TSC and NF1 cohorts.

Because of the sparsity of these syndromes, it is challenging to establish evidence-based guidelines, especially in terms of treatment. Patients with PNETs associated with germline mutations are underrepresented in phase III trials of drugs used in sporadic PNETs (21, 22). We have data on pharmacotherapy in patients with PNETs and MEN1 or VHL, but mostly from case reports or trials that assessed very small groups of patients (21–27). Despite the great interest in the topic, still, the surgical treatment of PNETs in MEN1/VHL remains controversial (9, 14, 28–30). These factors determine the need for further research, especially in terms of prognostic factors in PNETs and the impact of different treatment approaches on survival.

The heterogeneous disease course with a growing number of genetic mutations and lack of evidence from well-established studies concerning the treatment in hereditary PNETs should lead to careful adaptations of therapeutic schemes used in sporadic PNETs. Nevertheless, these schemes of sporadic endocrine tumors have been used in such cases (31).

Direct comparisons of sporadic and hereditary PNETs were performed on small groups of patients; thus, we assumed that larger groups might reveal additional information about the differences and prognostic factors that could result in different management decisions. From our previous work (32), we know that the Polish population of patients with MEN1 syndrome differs from previously described European and Asian populations, especially within PNETs. For this reason, we presume that these differences could also be present in comparison to sporadic PNETs in the Polish population. Moreover, some authors suggest the presence of genotype–phenotype correlations and prognostic factors in MEN1-related PNET (33–35) and VHL-related PNETS (9, 36, 37), which implies the need for further evaluation in these areas.

Because of all the above-mentioned reasons, we performed a comprehensive assessment of Polish patients with PNETs associated with germline mutations (GpNETs) and compared this group with patients with sporadic PNETs (SpNETs) and evaluated prognostic factors of metastases and survival in both groups. We also attempted to establish a genotype–phenotype correlation in the GpNET group.

## Materials and Methods

We retrospectively analyzed the medical data of patients with sporadic and hereditary PNETs treated from January 2004 to February 2020 in the following departments:

- Department of Nuclear Medicine and Endocrine Oncology, Maria Sklodowska-Curie National Research Institute of Oncology, Gliwice Branch, Gliwice, Poland
- Department of Internal Diseases and Endocrinology, Medical University of Warsaw, Warsaw, Poland

We searched our databases for patients with a genetic syndrome with described susceptibility to the development of PNET: MEN1, VHL, NF1, and TSC. All the familial syndromes were diagnosed according to present guidelines (38–41). We did not find patients with NF1 or TSC and diagnosed PNET in our databases, so our analysis applied only to patients with MEN1 and VHL. We excluded patients without confirmed germline mutations of *MEN1* and *VHL* genes. We also excluded these patients (with MEN1 or VHL phenotype but without confirmed mutations) from the analyzed group of sporadic PNETs.

The diagnosis of PNET was based on histological confirmation (tumor biopsy or post-surgical pathological sample) or radiological findings (typical presentation in computed tomography (CT) or magnetic resonance imaging (MR) and increased pathological uptake in Galium-68 positron emission tomography/CT) if no histological sample was available. Tumor grading was based on present European Neuroendocrine Tumor Society (ENETS) guidelines, and clinical staging was based on the 8^th^ Edition (2017) of TNM for PNETs from the American Joint Committee on Cancer (AJCC).

We assessed the following variables: age, sex, time of observation, age at PNET diagnosis, tumor features (grade, maximal tumor diameter in CT/MR or histological examination, and the number of tumors—multi/unifocal), clinical staging according to AJCC’s 8^th^ edition, site of metastases, type of treatment, additional diseases (including other neoplasms), and types of mutations in the case of MEN1 and VHL patients. Other neoplasms were defined as neoplasms other than typical for the particular hereditary syndrome/disease, described in the diagnostic criteria. Neuroendocrine tumors were accounted separately.

### Genetic Analysis

The study of the *VHL* and *MEN1* genes was performed in the Laboratory of Molecular Diagnostics and Functional Genomics at the Department of Nuclear Medicine and Endocrine Oncology and at the Department of Genetic and Molecular Diagnostics of Cancer. The *VHL* gene was analyzed by Sanger sequencing and targeted next-generation sequencing (NGS), while the *MEN1* gene analysis was performed by HRM (High-Resolution Melting), Sanger sequencing, and targeted NGS. Analysis of large deletions and duplications of the *MEN1* gene was performed using the MLPA (Multiplex Ligation-dependent Probe Amplification) technique. When genetic alterations (mutations, deletions, insertions, or duplications) were detected, first degree relatives were also included in genetic testing. Mutation types were divided by their effect on the DNA sequence into: missense, nonsense, splice-site, duplication, frameshift + STOP codon, whole exon deletion. Nonsense mutations were characterized by the substitution of a single base pair that leads to the appearance of the premature STOP codon which results in shortened and likely non-functional, protein. Frameshift + STOP codon mutations were characterized by deletion and/or insertion of a few nucleotides that resulted in change in DNA sequence leading to appearance of the premature STOP codon which may also result in shortened and likely non-functional, protein.

The details concerning genetic testing are presented in **Supplementary File 1**.

### Statistical Analysis

Categorical variables were summarized as frequencies and percentages, and continuous variables were shown as median values with interquartile ranges (25 to 75%, IQR 25–75) and as mean values with standard deviation ranges as well with min/max ranges, unless otherwise stated. Pairwise comparisons between patient subgroups were performed by Fisher’s exact test for categorical variables, and odds ratio was calculated. For continuous variables, comparisons between two groups were determined using Wilcoxon rank sum test, and for more than two patient subgroups, comparisons were performed by Kruskal–Wallis H test. The cut-off points for SpNET, GpNET, non-functional SpNET (NF-SpNET), and non-functional GpNET (NF-GpNET), as well as sensitivity and specificity, were determined by metastases status (present or not) using receiver-operating characteristic (ROC) curves. Youden Index was used to determine the cut-off point with the highest combination of sensitivity and specificity. Overall survival (OS) was defined as the time from diagnosis until death from pancreatic neuroendocrine tumor, or the last known date alive. Survival curves were plotted with the Kaplan–Meier method and compared using the log-rank test. Point-biserial correlation coefficient was assessed to examine the correlation between variables. Factors associated with survival endpoint were investigated by Cox proportional hazards model. All analyses were performed using R software package version 4.0.1 (R Foundation for Statistical Computing, http://www.r-project.org). A two-sided p-value <0.05 was considered statistically significant, and p-value <0.10 was considered close to statistical significance.

## Results

**Figure 1:**
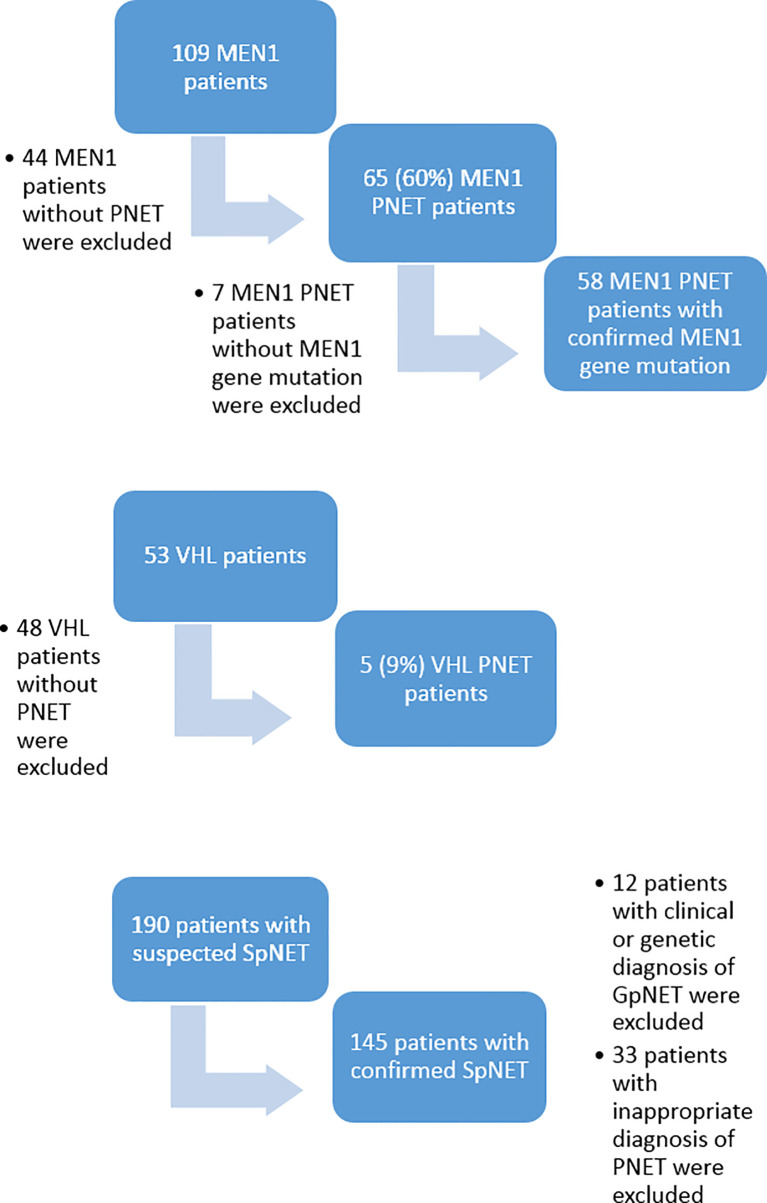
The flowchart of the recruitment process. MEN1, Multiple Endocrine Neoplasia type 1; VHL, Von Hippel–Lindau disease; PNET, pancreatic neuroendocrine tumor; SpNET, sporadic pancreatic neuroendocrine tumor.

Out of 109 patients with a clinical diagnosis of MEN1, 65 (60%) were diagnosed with PNET, although only 58 of them had confirmed germline *MEN1* gene mutation. Out of 53 patients with VHL, only five (9%) were diagnosed with PNET. Eventually, 63 patients met the inclusion criteria for the GpNET group. This group comprised 58 patients with MEN1-related PNET and five patients with VHL-related PNET. Out of 190 patients with sporadic PNET according to the medical database, 12 were excluded because of clinical or genetic diagnoses of MEN1 or VHL, and 33 were excluded because of inappropriate diagnoses of PNET. Eventually, 145 patients with SpNET were included in the analysis. **Figure 1** presents the flowchart of the recruitment process.

### General Information About the Groups

The patients in the GpNET group were younger (mean age 46.7 ± 13.8 years *vs*. 64.0 ± 12.3 years; p < 0.001) and were diagnosed with PNET earlier (41.4 ± 14.9 years *vs*. 59.7 ± 12.6 years; p < 0.001). There was no difference in sex distribution between the groups. Patients in the SpNET group were significantly more often affected by additional diseases [cardiovascular (OR = 2.963), diabetes (OR = 6.218), and kidney disease (OR = 4.599)], but there were no significant differences in terms of the prevalence of other neoplasms or other neuroendocrine tumors. Median time of follow-up was longer (p = 0.005) in the GpNET group (61 months; IQR 30.8–92.6) than in the SpNET group (39.5 months; IQR 17.8–73.3). **Table 1** presents all the general information about the groups.

**Table 1:** The general information about the groups.

	GpNET n = 63 (100%)	SpNET n = 145 (100%)	P value
Sex:			0.76
Male	25 (40%)	62 (43%)	
Female	38 (60%)	83 (57%)	
Deaths	4 (6%)	24 (17%)	**0.049**
Age [years] mean ± SD]	46.7 (± 13.8)	64.0 (± 12.3)	**<0.001**
Age at diagnosis [years] mean ± SD	41.4 (± 14.9)	59.7 (± 12.6)	**0.005**
Cardiovascular diseases	10 (16%)	52 (36%)	**0.005**
Diabetes	4 (6%)	43 (30%)	**<0.001**
Chronic Kidney Disease	2 (3%)	19 (13%)	**0.04**
Other neoplasms (excluding NET)	16 (25%)	30 (21%)	0.47
Other NET	5 (8%)	3 (2%)	0.06
Syndromes/diseases:		n/a	n/a
MEN1	58 (92%)		
VHL	5 (8%)		

GpNET, hereditary pancreatic neuroendocrine tumor; SpNET, sporadic pancreatic neuroendocrine tumor; MEN1, Multiple Neuroendocrine Neoplasia type 1; VHL, Von Hippel–Lindau disease The statistically significant p values are bolded.

Upon univariate analysis, we found that patients in the GpNET group with functional tumors (F-GpNET) were diagnosed earlier (30.1 ± 14.8 years *vs*. 44.8 ± 13.1 years, p = 0.002) than those with NF-GpNET. We did not observe this in the SpNET group. The age at diagnosis did not differ significantly with sex, stage, grade, and death rate in both GpNET and SpNET groups.

### Tumor Characteristics

**Figure 2:**
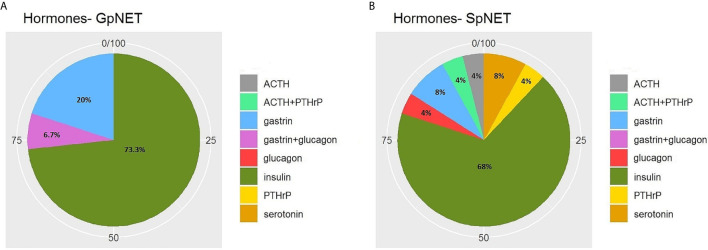
The diversity of the secreted hormones in GpNET **(A)** and SpNET **(B)**. GpNET, hereditary pancreatic neuroendocrine tumor; SpNET, sporadic pancreatic neuroendocrine tumor; ACTH, Adrenocorticotropic hormone; PTHrP, Parathyroid hormone-related protein.

**Figure 3:**
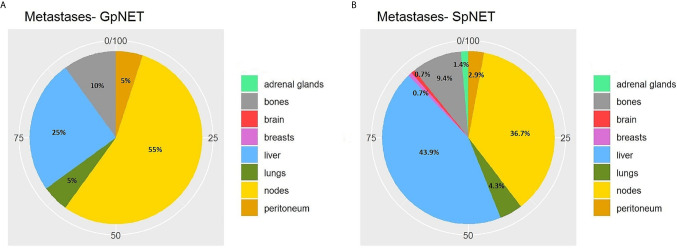
The distribution of metastases in GpNET **(A)** and SpNET **(B)**. GpNET, hereditary pancreatic neuroendocrine tumor; SpNET, sporadic pancreatic neuroendocrine tumor.

The two groups differed significantly in terms of the basis for PNET diagnosis (p < 0.001). In the GpNET group, it was most often typical radiological findings, whereas in the SpNET group, histological confirmation outweighed. The tumors in the GpNET group were smaller (mean tumor diameter 2.44 cm ± 1.92 *vs*. 3.88 cm ± 2.8; p < 0.001) and more often multifocal than those in the SpNET group (OR = 49.636). We did not observe any significant differences in terms of hormonal function. **Figure 2** presents the diversity of the secreted hormones in both groups. We observed metastases more often in the SpNET group [55 *vs*. 22%, p = <0.001 (OR = 4.355)], although there was no significant difference in terms of the location of the metastases. **Figure 3** presents the distribution of metastases. Additionally, patients in the SpNET group were assigned more often to the advanced stages [*i.e.*, III and IV (OR = 5.211)] than those in the GpNET group, although there were no significant differences in terms of grade. **Table 2** summarizes all the tumor characteristics of both groups.

**Table 2:** The tumor characteristics of both groups.

	GpNET n = 63 (100%)	SpNET n = 145 (100%)	P value
Base for PNET diagnosis:			**<0.001**
Pathology	38 (60%)	128 (88%)	
Radiology	25 (40%)	17 (12%)	
Tumor diameter [cm] mean ± SD	2.44 (± 1.92)	3.88 (± 2.8)	**<0.001**
Number of tumors:	n = 61 (100%)		**<0.001**
Single	22 (36%)	140 (97%)	
Multifocal	39 (64%)	5 (3%)	
Mean tumor number mean ± SD	4.9 (± 3.7)	n/a	n/a
Hormonal status:	n = 62 (100%)		0.25
F-PNET	15 (24%)	25 (17%)	
NF-PNET	47 (76%)	120 (83%)	
Metastases	n=59 (100%)		**<0.001**
	13(22%)	80 (55%)	
Grade	n = 26 (100%)	n = 127 (100%)	
G1	15 (58%)	63 (50%)	0.52
G2	11 (42%)	55 (43%)	1
G3	0 (0%)	9 (7%)	0.36
Stage	n = 59 (100%)	n = 136 (100%)	
I	24 (41%)	33 (24%)	0.026
II	22 (37%)	22 (16%)	**0.003**
III	7 (12%)	13 (10%)	0.62
IV	6 (10%)	68 (50%)	**<0.001**
I+II	46 (78%)	55 (40%)	**<0.001**
III+IV	13 (22%)	81 (60%)	

GpNET, hereditary pancreatic neuroendocrine tumor; SpNET, sporadic pancreatic neuroendocrine tumor; F-PNET, functional pancreatic neuroendocrine tumor; NF-PNET, non-functional pancreatic neuroendocrine tumor. The statistically significant p values are bolded.

Upon univariate analysis, we observed a significantly increased metastatic rate with growing tumor diameter in the SpNET group (R = 0.5; p < 0.001) and the GpNET group (R = 0.35; p = 0.007). The same correlation was observed in NF-SpNET patients (R = 0.44; p < 0.001) and NF-GpNET patients (R = 0.51; p < 0.001). We established cut-off points for tumor diameter, above which the metastatic rate increased significantly. They are 4 cm for both GpNET and NF-GpNET, 2.3 cm for SpNET, and 1.9 cm for NF-SpNET.

Moreover, we showed that with increasing grade, the metastatic rate increased significantly (p = 0.038 for the GpNET group and p < 0.001 for the SpNET group), and only in the case of SpNET, an increase in tumor diameter was observed (p = 0.007). We also observed a tendency toward decreased rate of functional tumors with increasing grade and stage in both groups.

### Treatment

We found that patients with SpNET more often underwent surgery [57 *vs*. 44%; p = 0.01 (OR = 1.673)], pharmacotherapy [48 *vs*. 27%; p = 0.006 (OR = 2.457)], peptide receptor radionuclide therapy (PRRT) [23 *vs*. 3%; p < 0.001 (OR = 9.342)], and chemotherapy [13% vs. 2%; p = 0.009 (OR = 9.349)]. No significant differences in radiotherapy were noted.

Recurrence after surgery tended to be more frequent in the GpNET cohort (p = 0.056).

**Table 3** presents the data regarding treatment.

**Table 3:** The data regarding treatment.

	GpNET n = 63 (100%)	SpNET n = 145 (100%)	P value
Surgery:	28/63 (44%)	83/145 (57%)n = 80 (100%)	**0.01**
tumor enucleation	8/28 (28.6%)	20/80 (25%)	0.8
distal resection	17/28 (60.7%)	50/80 (62.5%)	1
total pancreatectomy	3/28 (10.7%)	10/80 (12.5%)	1
Recurrence after surgery	13/28 (46%)	21/83 (25%)	0.056
Pharmacotherapy:	17/63 (27%)	69/145 (48%)	**0.006**
somatostatin analogs	16/17(94%)	49/69 (71%)	0.059
everolimus/sunitinib	0/17(0%)	2/69(3%)	1
combination	1/17(6%)	18/69 (26%)	0.1
PRRT treatment:	2/63 (3%)	34/145 (23%)	**0.0002**
Lu177	0/2 (0%)	13/34 (38%)	0.52
Y90	2/2 (100%)	6/34 (18%)	**0.04**
Combination	0/2 (0%)	15/34 (44%)	0.5
Chemotherapy	1/63 (2%)	19/145 (13%)	**0.009**
Radiotherapy	0/63 (0%)	9/145 (6%)	0.06

GpNET, hereditary pancreatic neuroendocrine tumor; SpNET, sporadic pancreatic neuroendocrine tumor; PRRT, peptide receptor radionuclide therapy; Lu177, Lutetium 177; Y90, Yttrium 90. The statistically significant p values are bolded.

Univariate analysis demonstrated that the recurrence rate in the SpNET group was higher in patients with higher grade (p = 0.004), higher stage (p < 0.001). Additionally, patients with recurrence tended to have bigger tumor diameter (p = 0.03). In GpNET higher recurrence rate was observed in patients with higher grades, but no statistical significance was achieved. Type of surgical intervention did not have an influence on recurrence in both groups.

### Survival

During the 140 month observation period, patients in the SpNET group experienced significantly more deaths [17 *vs*. 6%, p = 0.049 (OR = 2.926)] and shorter survival (73.9 ± 7.2 months *vs*. 94.7 ± 3.1 months; p = 0.015) than those in the GpNET group. Upon univariate analysis, survival was associated with age at diagnosis, sex, grade, stage, tumor diameter, rate and localization of metastases, type of treatment, and comorbidities. The worse survival in the SpNET group was determined by the following factors: age at diagnosis older than 60 years, higher grade, higher stage, bigger tumor diameter, present metastases, concomitant hepatic and nodal metastases, only pharmacological treatment in comparison to surgery, and absence of other neoplasms. Additional analysis of the subgroup of patients with SpNET diagnosed after the age of 60 showed a significantly lower survival in patients with no other neoplasms but no impact of comorbidities (cardiovascular disease, diabetes, and chronic kidney disease) on survival. In the GpNET group, the presence of other neoplasms signified worse survival. Regarding tumor diameter, a cut-off point of >2.3 cm in the SpNET group implied worse survival. In the analysis of the subgroups with non-functional tumors, there was no significant difference in terms of tumor size and survival in both the GpNET and SpNET groups. As no significant difference was also observed in GpNET with regard to cut-off established by ROC method, we analyzed the impact of arbitrarily established cut-off of 2 cm. In this analysis tumors with diameter >2 cm implied worse survival.

**Figure 4:**
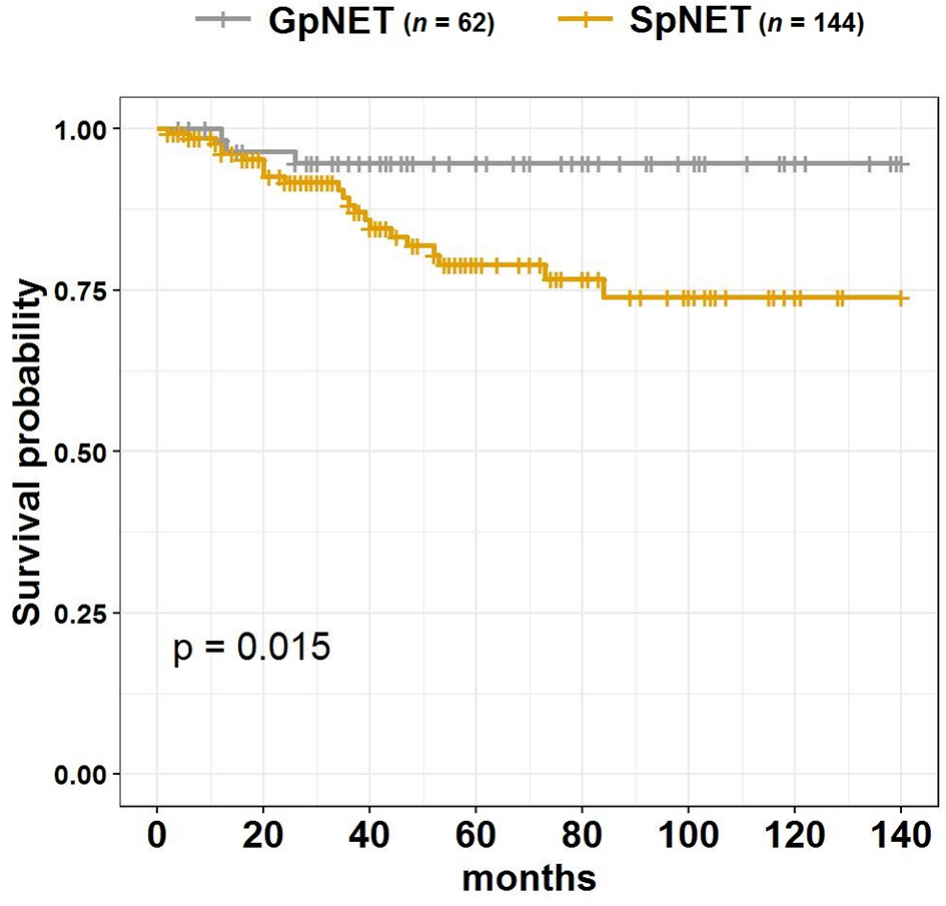
The Kaplan–Meier curves of overall survival (OS) in GpNET and SpNET. GpNET, hereditary pancreatic neuroendocrine tumor; SpNET, sporadic pancreatic neuroendocrine tumor.

**Figure 5:**
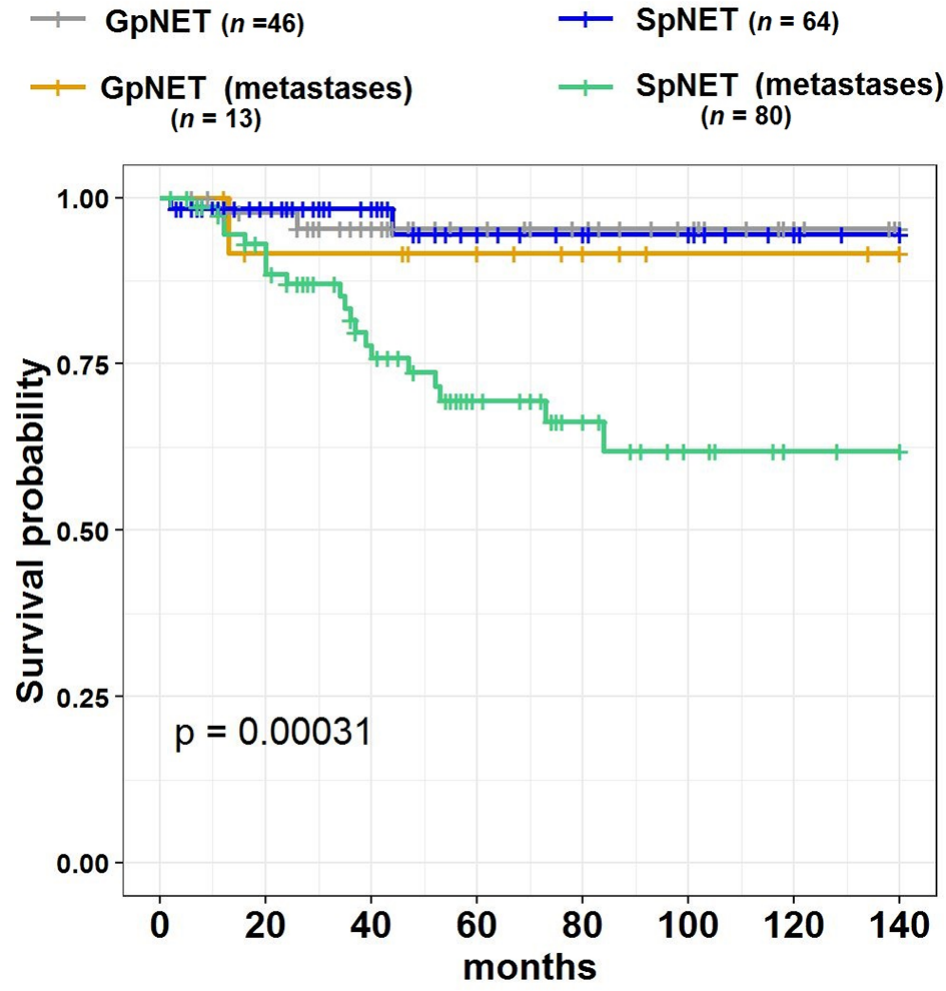
The Kaplan–Meier curves of overall survival (OS) regarding the presence of metastases in GpNET and SpNET. GpNET, hereditary pancreatic neuroendocrine tumor; SpNET, sporadic pancreatic neuroendocrine tumor.

**Figure 6:**
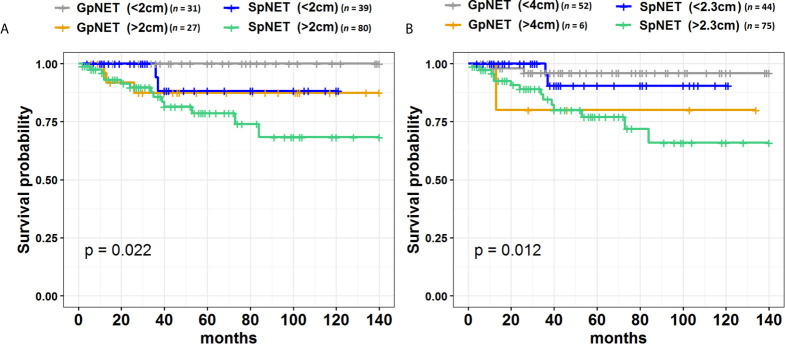
The Kaplan–Meier curves of overall survival (OS) regarding the tumor diameter cut-off in GpNET and SpNET. Panel **(A)** presents the arbitrarily established cut-off point of 2 cm, and panel **(B)** presents the cut-off points established by ROC method. GpNET, hereditary pancreatic neuroendocrine tumor; SpNET, sporadic pancreatic neuroendocrine tumor.

**Figure 4** presents the Kaplan–Meier curves of overall survival (OS) in the GpNET and SpNET groups. **Figure 5** presents the Kaplan–Meier curves of OS regarding the presence of metastases. **Figure 6** presents the Kaplan–Meier curves of OS regarding the tumor diameter with arbitrarily established cut-off (A) and cut-off established by ROC method (B).

A comparison of both groups revealed that the following features differentiate them in terms of survival: age at diagnosis, sex, grade, stage, hormonal status, presence of other neoplasms, and diseases. Patients with GpNET, patients of the female sex, diagnosed before the age of 40, with a functional tumor, with no other neoplasms, and additional diseases had better survival than those with SpNET having the same features.

### Genetics

**Figure 7:**
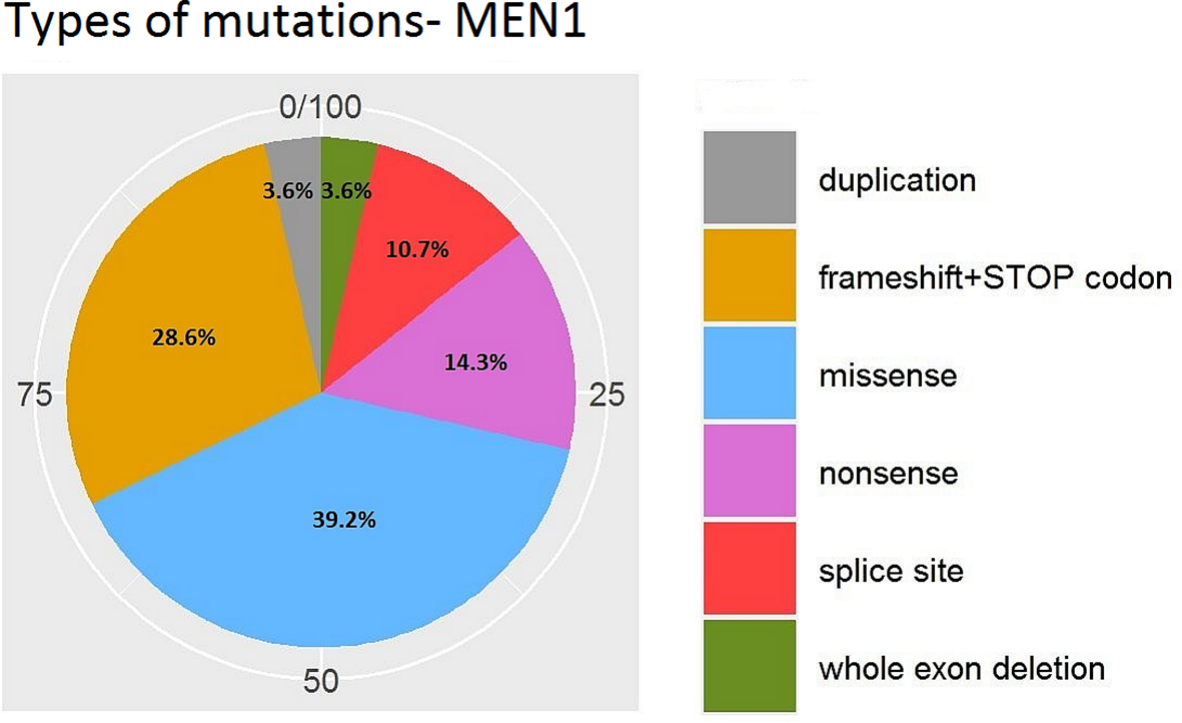
Types of mutations in MEN1-related PNETs. MEN1, Multiple Endocrine Neoplasia type 1; PNET, pancreatic neuroendocrine tumor.

**Figure 8:**
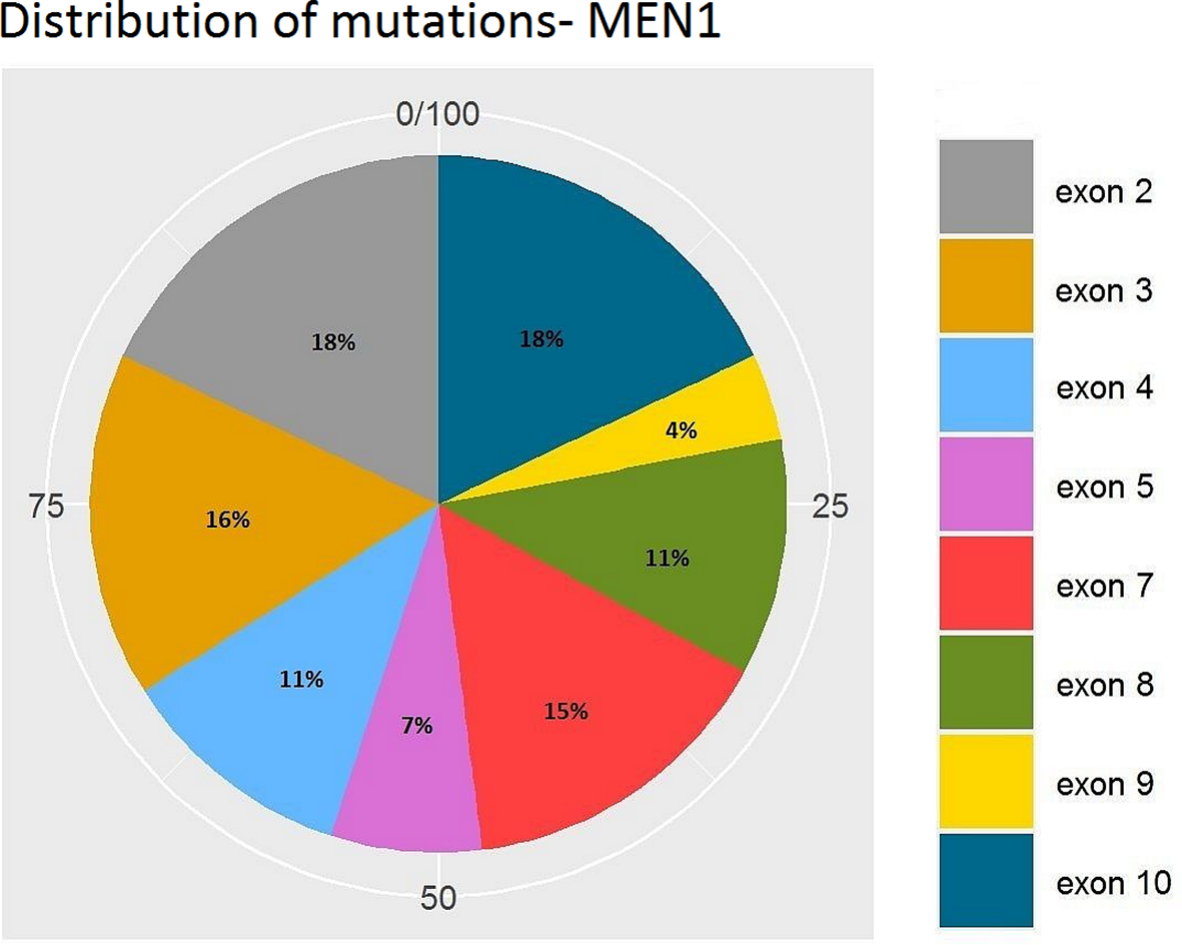
Distribution of mutations along exons of the *MEN1* gene. MEN1, Multiple Endocrine Neoplasia type 1.

**Figures 7** and **8** present the distributions of the types of mutations and involved exons in patients with MEN1-related PNETS. **Table 4** presents genetic data of VHL patients. Because of the small number of VHL patients, we performed univariate analysis only for MEN1 patients. In this group, we observed a strong negative correlation between frameshift + STOP (R = −0.958; p = 0.042) or splice-site mutations (R = −1; p < 0.001) and stage. With increasing stage, the rate of the above-mentioned mutations decreased. Similarly, with increasing grade, the rate of missense mutation decreased (R = −0.996; p = 0.05). We found that carriers of mutations in exon 5 were diagnosed with PNETs significantly earlier than those who carried mutations in exons 2, 3, 4, 8, and 10. Additionally, patients with mutations in exon 2 tended to have metastases more frequently [p = 0.04 (OR = 4.857)]. The type of mutation did not differ with the metastatic rate and functional status. Involved exons did not differ with the grade, stage, and functional status.

**Table 4:** The genetic data of VHL patients.

VHL patients	VHL gene mutation	Type of mutation	exon
Patient 1	c.375A>C, p.(His125Pro)	missense	2
Patient 2	c.163_164insG	frameshift + STOP codon	1
Patient 3	c.500G>A, p.(Arg167Gln)	missense	3
Patient 4	c.463 +2T>G	splice site	n/a (intron mutation)
Patient 5	c.407T>C, p.(Phe136Ser)	missense	2

### Multivariable Analysis

Upon multivariable analysis, we assessed the impact of the following groups of variables in both SpNET and GpNET on survival separately:

- group 1: age, age at diagnosis, sex, tumor diameter, and other diseases
- group 2: tumor diameter, metastases, grade, and hormonal status
- group 3: tumor diameter, metastases, surgery, pharmacotherapy, and recurrence after surgery

Among the variables from the first group in GpNET, older age, bigger tumor size, and the presence of other neoplasms and neuroendocrine tumors were observed more often in patients with a higher mortality rate. In the same group of variables, only older age at diagnosis appeared to be significantly associated with a higher mortality rate in the SpNET group. In the second group of variables, only the presence of metastases in the SpNET group was observed more often in patients with a higher mortality rate. In group 3, no significant differences were found in the SpNET group. After selection, no patients from the GpNET group were included for this analysis. **Tables 5**–**7** present the results of the multivariable analysis.

**Table 5:** The multivariable analysis of impact of variables from the first group on overall survival.

Endpoint	Variable	HR (95% CI)	P value
GpNET			
OS	**Age**	**1.11 (1**–**1.23)**	**0.05**
	Age at diagnosis	0.95 (0.88–1.05)	0.31
	Sex	2.19 (0.2–24.34)	0.52
	**Tumor diameter**	**2.27 (1.62**–**3.19)**	**0.001**
	**Other neoplasms**	**59.3 (4.88**–**720.85)**	**0.001**
	**Other NET**	**17.33 (1.56**–**192.49)**	**0.02**
	Cardiovascular disease	<0.001 0.00–∞	1
SpNET			
OS	Age	0.92 (0.83–1.02)	0.1
	**Age at diagnosis**	**1.13 (1.03**–**1.24)**	**0.01**
	Sex	2.86 (0.96–8.49)	0.06
	Tumor diameter	1.07 (0.89–1.28)	0.5
	Other neoplasms	1.16 (0.02–1.37)	0.1
	Cardiovascular disease	0.38 (0.10–1.52)	0.17
	Diabetes	1.44 (0.42–4.98)	0.56
	Kidney disease	1.89 (0.46–7.72)	0.38

GpNET, hereditary pancreatic neuroendocrine tumor; SpNET, Sporadic pancreatic neuroendocrine tumor. The statistically significant p values are bolded.

**Table 6:** The multivariable analysis of impact of variables from the second group on overall survival.

Endpoint	Variable	HR (95% CI)	P value
GpNET			
OS	Tumor diameter	38.93 (0.00–∞)	1
	Metastases	9.01 (0.00–∞)	1
	Grade	97.71 (0.00–∞)	1
	Functional tumor	0.28 (0.00–∞)	1
SpNET			
OS	Tumor diameter	0.92 (0.77–1.14)	0.53
	**Metastases**	**1.13 (1.5**–**124.69)**	**0.02**
	Grade	2.86 (0.39–2.37)	0.04
	Functional tumor	1.07 (0.65–9.13)	0.19

GpNET, hereditary pancreatic neuroendocrine tumor; SpNET, sporadic pancreatic neuroendocrine tumor. The statistically significant p values are bolded.

**Table 7:** The multivariable analysis of impact of variables from the third group on overall survival.

Endpoint	Variable	HR (95% CI)	95 confidence intervals
SpNET			
OS	Tumor diameter	1.09 (0.78–1.51)	0.62
	Metastases	<0.001 (0.00–∞)	1
	Surgical treatment	<0.001 (0.00–∞)	1

The most significant univariate correlations are presented in **Supplementary File 2**.

## Discussion

The main goal of our study was to compare the clinical data of patients with GpNET to those of patients with SpNET and to assess prognostic factors in both groups of patients. Additionally, an attempt to perform a genotype–phenotype analysis in the GpNET group was made. Surprisingly, despite much attention on the subject of PNET, only a few direct comparisons on small groups of patients were published. We assume that differences based on comparisons between different studies evaluating GpNET and SpNET separately could be prone to bias. Additionally, these studies often either analyze only a particular subgroup (*e.g.*, NFpNET) or do not exclude patients with MEN1 or VHL in the case of “PNET” analysis, which makes it less significant when compared. To the best of our knowledge, we managed to directly compare one of the biggest cohorts of patients diagnosed with GpNET and SpNET.

Some of our results are consistent with previously published data (13, 14, 19, 20, 42, 43). However, we also managed to provide new data, especially regarding the prognostic factors of metastases and survival and genotype–phenotype correlations.

We found only one study that had reported a larger tumor diameter in patients with SpNET in comparison with GpNET (44). Other studies showed a similar trend, but without statistical significance (19, 20, 45) or did not compare this feature (9, 13). Our results indicate that GpNETs have significantly smaller tumors than SpNETs, which could be associated with the higher rate of more advanced stages and higher tumor grades among patients with SpNET.

Although the multifocality of tumors had been suggested as a typical feature of GpNET, it was shown that in VHL-related PNET a majority of tumors are single (9, 13), which was also observed in our cohort (60% unifocal). This suggests that multifocality cannot be treated as a marker of hereditary PNET, at least regarding VHL.

We observed no significant difference in the rate of occurrence of functional tumors between the GpNET and SpNET groups and with no functional tumors in the VHL groups. Similar findings had been published by other authors (9, 19). Again, in a larger cohort of patients, we showed that insulinoma is the most frequent functional tumor in Polish patients with MEN1-related PNETs (32). The insulinoma predominance had been reported only in a Japanese cohort (46), whereas in other described cohorts, gastrinoma dominates (17, 47). The possible reason for this discrepancy had been suggested in our previous paper (32).

Our results provide promising data regarding tumor diameter cut-off values in terms of risk of metastases in both SpNET and GpNET groups. It is of major importance since it might add a significant argument in the discussion of the management of PNETs, where controversies still exist (14, 28, 29, 48–50).

A positive correlation between tumor size and metastatic rate in SpNET had previously been observed, and different cut-off (from 1.5 to 4.0 cm) points were established (48, 51–56). Unfortunately, there are significant differences in the methodology of these studies. Some of these authors included a small percentage of GpNET in their cohorts, whereas some did not evaluate this aspect (51–53). Others analyzed only a particular cohort, like NFpNET (48) or patients who underwent surgery (55). Also, differences in methodology regarding metastases were observed since some authors evaluated only the influence on nodal metastases (52, 56). These factors make it difficult to compare these studies with each other and with our results.

In the case of GpNET, similar results had been published by others (57) but mostly in particular cohorts—NFpNET (58, 59) or VHL (9, 36). Oleinikov et al. assessed a small group of patients where no genetic test was available for all the patients and almost half of those with genetic tests were negative for MEN1 mutations (57). Also, results showing no correlation were published (60–62), which could be a result of different populations (especially patients referred to surgery) that were assessed or a small sample size as in the study by Bartsch et al.

Although a trend toward a positive correlation between tumor diameter and metastatic rate in both groups seems undoubtful, cut-off points probably must be established more carefully regarding a particular cohort of patients. Moreover, we have to look for other factors favoring metastases since patients with small tumors and metastases are also described (63, 64), so tumor size cannot be the only factor determining management.

We found that overall survival was worse in the SpNET group than in the GpNET group. PNETs associated with MEN1 and VHL are believed to be indolent, although published results concerning survival are discordant. A few authors (20, 65, 66) showed that MEN1-related PNETs have better survival, whereas others (19, 67, 68) noted no significant difference in survival. Significant differences in analyzed groups, including the number of patients, duration of follow-up, method of genetic syndrome diagnosis, type of tumor (FpNET/NFpNET), and used treatment could play a role in these discrepancies. In a study by Demestier, the mortality rate in the VHL group was higher than that in the sporadic one. However, the study group was small and only one out of five deaths in the VHL group were PNET-related (14). Differences in survival between the different types of functional MEN1-related PNETs were observed (69). Since evaluated populations often differ in terms of the rate of particular types of functional tumors, it might be assumed that it influences the overall survival of the group (70). Moreover, there have been significant changes in the management of Zollinger–Ellison syndrome and primary hyperparathyroidism, which were responsible for a significant mortality rate in MEN1 syndrome in the past (15, 16). The retrospective nature of research and the inclusion of a significant number of patients treated before the era of improvements and assessment of overall non-disease-related survival might cause considerable bias.

The variables associated with worse survival in SpNET in our study were also described by others (6, 71, 72), but the age of patients and metastases seem to be the most significant ones. Only patients’ age, tumor size, and additional neoplasm or NET were shown to be prognostic factors in the GpNET group despite the existence of many of such factors described before (9, 35, 69). It is probably the result of a larger group of patients analyzed in those studies.

We observed that tumor diameter over 2.3 cm in SpNET and 2.0 cm in GpNET led to worse survival. The same trend as for SpNET was observed in the NF-SpNET group. Similar results had been reported by others in SpNET (54, 55), although a lower threshold (2 cm) was used and only NFpNET was evaluated. No significant impact of tumor diameter on survival in this group was also reported, which could be due to the small number of patients and the short period of follow-up (63, 73, 74), different methodological approach (75), or differences in the FpNET/NFpNET ratio (72).

In the GpNET group, mostly NFpNET in the context of MEN1 syndrome was evaluated. In this subgroup, the negative impact of tumor size over 2 cm was described (35, 76–78). One study assessing survival in MEN1-related gastrinoma reported the same size of a pancreatic tumor as a negative prognostic factor, although in MEN1, mostly disseminated duodenal tumors are responsible for gastrinoma and the measured PNET is probably additional NFpNET (79, 80).

Interestingly, we found a completely opposite influence of other neoplasms on survival in SpNET and GpNET. An extended analysis of a subgroup of 30 patients with SpNET and other neoplasms revealed that 17 out of 20 patients (where information about the time of diagnosis of other neoplasms was available) had other neoplasms diagnosed before or during the same year as NET. Broad-spectrum imaging diagnostics during neoplasm diagnosis and further follow-up screening could lead to the earlier diagnosis of lower-stage NET, which could impact the survival rate. A similar assumption could be made in patients with additional diseases who probably sought medical advice more often, which is why their tumors could be diagnosed earlier. Opposite results in terms of neoplasm in GpNET could be a result of early screening in almost all the patients with the diagnosed syndrome, which decreases the above-mentioned influence of follow-up described in SpNET. Moreover, in MEN1 patients, other potentially lethal tumors are known to present more often than in general populations, which could also play a role in increased mortality (81). Names outlined that although a favorable survival was shown in patients with multiple metachronous primary tumors, these results are based on small populations and depend on the tumor site (82). Other studies suggest that the order of diagnosed neoplasms might influence the survival rate (83). However, other studies with different results can be found (84). Koo et al. assessed a different neoplasm for a shorter period of time, and the major impact of highly lethal lung and stomach cancer was observed.

Our analysis of the correlation between genetic and clinical data demonstrates that some genetic variants might influence the disease course. Carriers of frameshift with the STOP codon, splice-site, and missense mutations tended to have less advanced disease, while mutations in exon 2 of *MEN1* gene implied more advanced disease. Although no genotype–phenotype correlation is established in MEN1, one can find promising results in the literature (33, 34, 85). Previously published papers are consistent with our findings with regard to negative impact of mutations in exon 2 on disease course (59, 85). These observations were assessed on a small number of patients with a borderline significance (p = 0.04 in our data and p = 0.049 in Christakis paper), so there is a need for further research on larger cohorts of patients to confirm this.

The analysis of four patients with exon 5 revealed that 75% of them had insulinoma, and 50% of them were diagnosed during regular follow-up as family members of proband diagnosed with MEN1. These factors could have a significant impact on the observed correlation with early diagnosis and should be considered.

In summary, we found that Polish populations of GpNET and SpNET differ among many factors, not only when compared with each other but also when compared to other described populations. This should lead to different approach when managing GpNET or SpNET and encourage assessing each country/region populations of PNETs, having in mind possible differences and impact of a founder effect. We observed different, from previously described, cut-off points of tumor diameter that correlate with metastatic spread and worse survival in both GpNET and SpNET. In the light of other prognostic factors, controversies in therapy protocols (31), and studies showing a poor prognosis even in patients with tumor diameter below 2 cm (63, 64), we should base our therapeutic decisions on a more comprehensive approach rather than only analyzing tumor diameter impact. Ideally, it would result in the development of a simplified scale-based algorithm, comprising main prognostic factors. We demonstrated genetic changes (*i.e.* frameshift with the STOP codon, splice-site and missense mutations, or mutations in exon 2) which could have impact on the clinical course in MEN1-related PNETs. Confirming that in a larger cohort of patients could lead to the profiled therapy and follow-up according to genetic changes, which not only could improve patients’ quality of life, but probably also lower the cost of management.

Our study has some limitations. First, the retrospective design and the collection of data in different centers could lead to some bias. Still, we managed to comprehensively assess a large group of GpNET and SpNET patients, which is a difficult task considering the rarity of the disease and would be hardly possible in a prospective manner. Second, the GpNET group consisted mostly of MEN1 patients, and the results might not apply to VHL-, TSC-, and NF1-related PNETs. Nevertheless, we assume that the rarity of PNET in the rest of the syndromes suggests that these patients would not change the big picture of GpNET if analyzed together. Third, we assessed only the overall survival, not the disease-related one. In the light of the favorable impact of additional diseases and neoplasms in SpNET, we could assume that deaths were mostly associated with PNET.

## Conclusion

We showed that patients with SpNET and GpNET differ significantly, and one should consider this difference when deciding on the management. The proposed prognostic factors should be confirmed in larger cohorts of patients, and, by then, applied carefully with an individualized approach to every patient. International collaboration is needed to establish any reliable genotype–phenotype correlation in GpNET.

## Data Availability Statement

The datasets presented in this article are not readily available because data is anonymized, and according to our rules, available only for patient and treating physician. Requests to access the datasets should be directed to przemyslaw.soczomski@io.gliwice.pl.

## Ethics Statement

Ethical review and approval were not required for the study on human participants in accordance with the local legislation and institutional requirements. Written informed consent for participation was not required for this study in accordance with the national legislation and the institutional requirements.

## Author Contributions

PS—design of the study, data collection, data analysis, manuscript writing, and literature analysis. BJ-L—design of the study, data collection, and critical review. AK—statistical analysis and graphical presentation of results. AC—statistical analysis. SZ—data collection. NR—data collection. MO-W—genetic analysis and interpretation. AP—genetic analysis and interpretation. TB—data collection. BJ—critical review. All authors contributed to the article and approved the submitted version.

## Funding

AC was co-financed by the European Union through the European Social Fund (grant no. POWR.03.02.00–00–I029).

## Conflict of Interest

The authors declare that the research was conducted in the absence of any commercial or financial relationships that could be construed as a potential conflict of interest.
