# Supplementary material for: A Direct Comparison of Patients With Hereditary and Sporadic Pancreatic Neuroendocrine Tumors: Evaluation of Clinical Course, Prognostic Factors and Genotype–Phenotype Correlations
Source: Front Endocrinol (Lausanne). 2021 May 28;12:681013. doi: 10.3389/fendo.2021.681013 (PMC8194819; doi:10.3389/fendo.2021.681013)
Supplement: Supplementary file 1 [file Table_1.doc]

DNA isolation:

Genomic DNA was extracted from peripheral blood leukocytes. DNA isolation was performed with the Genomic Maxi AX DNA isolation kits (A&A Biotechnology, Gdynia, Poland) and the Maxwell RSC Blood DNA Kit (the latter with the use of Maxwell RSC Instrument; Promega, Madison, WI USA) accordance to the manufacturer's recommendations. Isolated DNA was assessed for the quantity, quality and purity with the NanoDrop ND1000 (Thermo Fisher). Samples analyzed with NGS were also assessed with the Qubit 2.0 (Thermo Fisher) using the dsDNA HS Assay Kit (Thermo Fisher).

Detection of *VHL* and *MEN1* gene mutations by Sanger sequencing:

The PCR reaction was performed using primers designed to cover the entire coding part of the gene (Supplementary Table 1). PCR conditions were adapted to recommendation for the HotStarTaq polymerase (QIAGEN, Hilden, Germany) and the particular pair of primers (annealing temperatures are given in Supplementary Table 1). PCRs were carried out on the TGradient instrument (Biometra, Jena, Germany).

PCR products were checked on a 2% agarose gel (Thermo Fisher) with the SYBR Safe DNA gel dye (Thermo Fisher) or with ethidium bromide (ICN Biomedical Inc, Erie, PA USA). Then PCR products were purified by SAP alkaline phosphatase (Shrimp Alkaline Phosphatase) and Exo I (Exonuclease I; Thermo Fisher) or the ExS-Pure enzyme kit (Nimagen, Nijmegen, The Netherlands) according to manufacturer's recommendations. Sanger sequencing was performed using BigDye Terminator v.1.1 Cycle Sequencing Kit (Thermo Fisher). Sequential electrophoresis was performed on the 3130xl Genetic Analyzer (Thermo Fisher). Obtained results were analyzed with the Sequencing Analysis v5.2 program. (Thermo Fisher).

Detection of *MEN1* gene mutation by HRM method.

The analysis was carried out with the use of the MeltDoctor HRM Master Mix kit (Thermo Fisher) according to the manufacturer's protocol. Primer pairs that were used for Sanger sequencing were used for the HRM procedure as well (Supplementary Table 1). The reaction was carried out on the 7900 HT Fast Real-Time PCR instrument (Thermo Fisher). Data analysis was performed using the High Resolution Melting program (Thermo Fisher).

Detection of *VHL* and *MEN1* genes mutations by next-generation sequencing:

Analysis of the *VHL* and *MEN1* genes by the next-generation sequencing method was implemented in 2017. NGS was performed using following kits: TrusSight Cancer (Illumina, San Diego, CA USA) or SeqCap EZ Choise (Roche, Basel, Switzerland) according to the manufacturer's protocols (TruSight Rapid Capture Reference Guide # 15043291v 0.1 Illumina or SeqCap EZ Hyper Cap Workflow Use's Guide v 1.0 Roche). The template for libraries synthesis was DNA at concentrations of 500 ng for the SeqCap EZ Choise kit (Roche) and 50 ng for the TrusSight Cancer kit (Illumina). Prepared libraries contained 12 to 14 pooled samples. The libraries were sequenced using the MiniSeq sequencer (Illumina) with the cardriges MiniSeq Mid Output Kit (300-cycles) (Illumina). The results were analyzed in the Variant Interpreter (Illumina). For the TruSight Cancer library, bioinformatic analysis (mapping, filtering of substandard quality readings, mutation detection) was performed on the Base Space platform according to the Burrows - Wheeler Aligner (BWA) Enrichment protocol. For the SeqCap EZ Choise library (Roche) data analysis was performed according to the Roche manufacturer's recommendations "How To Eveluate NimbleGene SeqCap EZ Target Enrichment Data". For mutation detection, the program GATK v.4.0.1.2 (Genome Analysis Tool Kit) HaplotypeCaller function was used.

Detection of large deletions and duplication of the *MEN1* gene by MLPA:

Analysis of large deletions and duplication of chromosome fragments was performed using the commercial SALSA-P017 MEN1 probe kit (MRC-Holland, Amsterdam, The Netherlands) according to the manufacturer's protocol. The electrophoresis was performed on the 3130xl Genetic Analyzer (Thermo Fisher) using HiFi Formamide (Thermo Fisher) and ROX500 size marker (Thermo Fisher). Analysis of the results was performed using the Coffalyser software (MRC-Holland).

Supplementary Table 1. Sequences of the *VHL* and *MEN1* gene primers:

| GENE | PRIMERS SEQUENCES | ANNEALING TEMPERATURE |
| --- | --- | --- |
| VHL 1FiR | F5’TAGCCAGGACGGTCTTGAT3’; R5’CGTACAAATACATCACTTC3’ | 59.1°C |
| VHL 2FiR | F5’GGACGGTCTTGATCTCCTG3’; R5’AGCCCAAAGTGCTTTTGAG3’ | 62°C |
| VHL 3FiR | F5’CTGTCACTGAGGATTTGGTT3’; R5’TAAGCAATGGTGCCTATTTT3’ | 57.2°Cx14 50.2°Cx19 |
| MEN1 2A | 2a *F5’* GGGCGGGTGGAACCTTAG3’; R5’AAGGAAAGGAGCACCAGGTC3’ | 60.0°C |
| MEN1 2B | 2b F5’CCCAGAAGACGCTGTTCC3’; R5’RATAGAGGGCGGCGATGATAG3’ | 59.9°C |
| MEN1 2C | 2c F5’GTCATCCCTACCAACGTTCC3’; R5’RATCGGAGACCTTCTTCACCA3’ | 58.3°C |
| MEN1 2D | 2d F5’CTCTATGCCCGCTTCACC3’; R5’CCATGGAGGGTTTTGAAGAA3’ | 60.8°C x14 53.8°C x19 |
| MEN1 3 | 3 F5’CCTTTCCCCATGTTAAAGCA3’; R5’GGTGGCTTGGGCTACTACAG3’ | 61.0°C |
| MEN1 4 | 4 F5’TGGGCCATCATGAGACATAA3’; R5’GGTCCCACAGCAAGTCAAGT3’ | 61.0°C |
| MEN1 5i6 | 5 and 6 F5’GCTAAGGACCCGTTCTCCTC3’; R5’CAGCCACTGTTAGGGTCTCC3’ | 62.0°C |
| MEN1 7 | 7 F5’GGCTGCCTCCCTGAGGAT3’; R5’AGTCCTGGACGAGGGTGGT3’ | 60.9°C |
| MEN1 8 | 8 F5’CTACCCCCGATGGTGAGAC3’; R5’GTGGGAGGCTGGACACAG3’ | 60.9°C |
| MEN1 9 | 9F5’TCTGCTAAGGGGTGAGTAAGAGA3’; R5’ACCACCTGTAGTGCCCAGAC3’ | 60.9°C |
| MEN1 10A | 10a F5’CAGAGCAGGGTCCTGGAGTT3’; R5’GGGGTCCTGACACTGCAC3’ | 60.9°C |
| MEN1 10B | 10b F5’CGCATAGTGAGCCGAGAGG3’; R5’TCTGGAAAGTGAGCACTGGA3’ | 62.0°C |
| MEN1 10C | 10c F5’CAGGGTGCAGTGTCAGGA3’; R5’TAGGGGTGGACACTTTCTGC3’ | 58.9°C |
| MEN1 10D | 10dF5’AAGATGAAGGGCATGAAGGA3’; R5’GGGAACCTAGGGTTTGGGTA3’ | 61.0°C |
