# Supplementary material for: A Direct Comparison of Patients With Hereditary and Sporadic Pancreatic Neuroendocrine Tumors: Evaluation of Clinical Course, Prognostic Factors and Genotype–Phenotype Correlations
Source: Front Endocrinol (Lausanne). 2021 May 28;12:681013. doi: 10.3389/fendo.2021.681013 (PMC8194819; doi:10.3389/fendo.2021.681013)
Supplement: Supplementary file 2 [file Table_2.docx]

Supplementary Table 2. The univariate correlations discussed in the manuscript. Abbreviations: MEN1 – Multiple Endocrine Neoplasia type 1; SpNET – Sporadic Pancreatic Neuroendocrine Tumor, GpNET – Hereditary Pancreatic Neuroendocrine Tumor, NF-SpNET – Non-functional SpNET; NF-GpNET – Non-functional GpNET.

| **Correlation comparisons** | **Group** | **Spearman correlation** | |
| --- | --- | --- | --- |
|  |  | **R** | ***p-value*** |
| metastatic rate vs tumor diameter | SpNET | 0.500 | < 0.001 |
| metastatic rate vs tumor diameter | GpNET | 0.350 | 0.007 |
| metastatic rate vs tumor diameter | NF-SpNET | 0.440 | < 0.001 |
| metastatic rate vs tumor diameter | NF-GpNET | 0.510 | < 0.001 |
| rate of frameshift + STOP vs stage | MEN1 patients | -0.958 | 0.042 |
| rate of splice-site mutations vs stage | MEN1 patients | -1.000 | < 0.001 |
| rate of missense mutation vs grade | MEN1 patients | -0.996 | 0.05 |
